# Supplementary material for: The cryo-EM structure of the bacterial flagellum cap complex suggests a molecular mechanism for filament elongation
Source: Nat Commun. 2020 Jun 25;11:3210. doi: 10.1038/s41467-020-16981-4 (PMC7316729; doi:10.1038/s41467-020-16981-4)
Supplement: Supplementary file 1 — Supplementary Information [file 41467_2020_16981_MOESM1_ESM.pdf]

Supplementary Information

The cryo-EM structure of the bacterial flagellum cap complex suggests a molecular mechanism for filament elongation

Al-Otaibi *et al*.

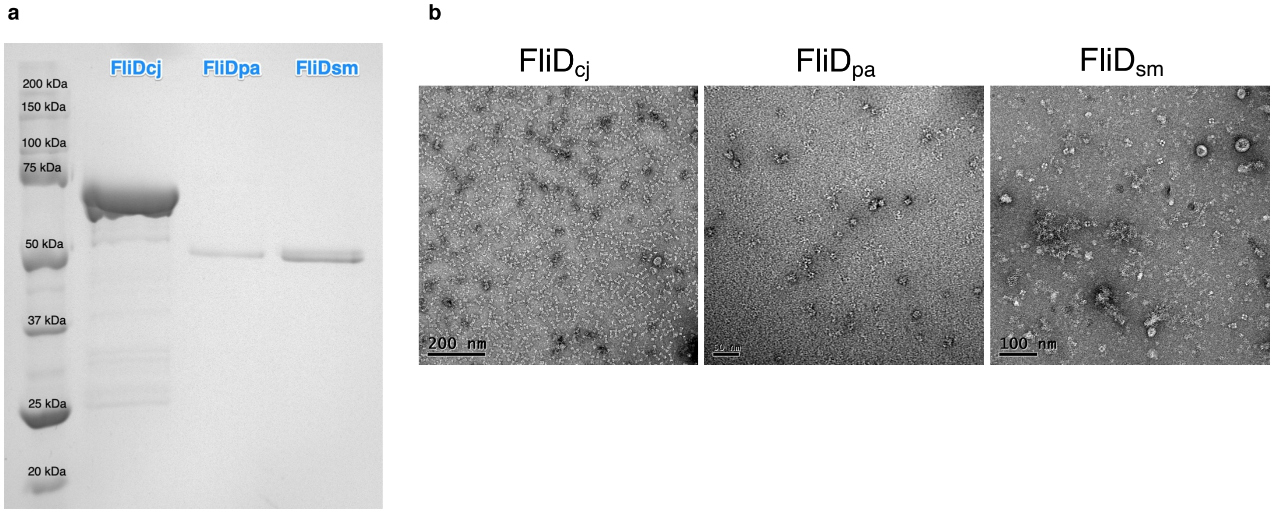

Supplementary Figure 1: Purification and oligomerization of recombinant FliD. (a) SDS-PAGE gel showing the purified recombinant $FliD_{cj}$ (70 kDa), $FliD_{pa}$ and $FliD_{sm}$ (50 kDa) proteins. **(b)** Negative stain of the samples in (a), diluted to ~ 0.1 mg/ml. $FliD_{cj}$ appears as well-distributed, homogeneous particles, while $FliD_{pa}$ and $FliD_{sm}$ form heterogeneous particles, with significant aggregation. Source data are provided as a Source Data file.

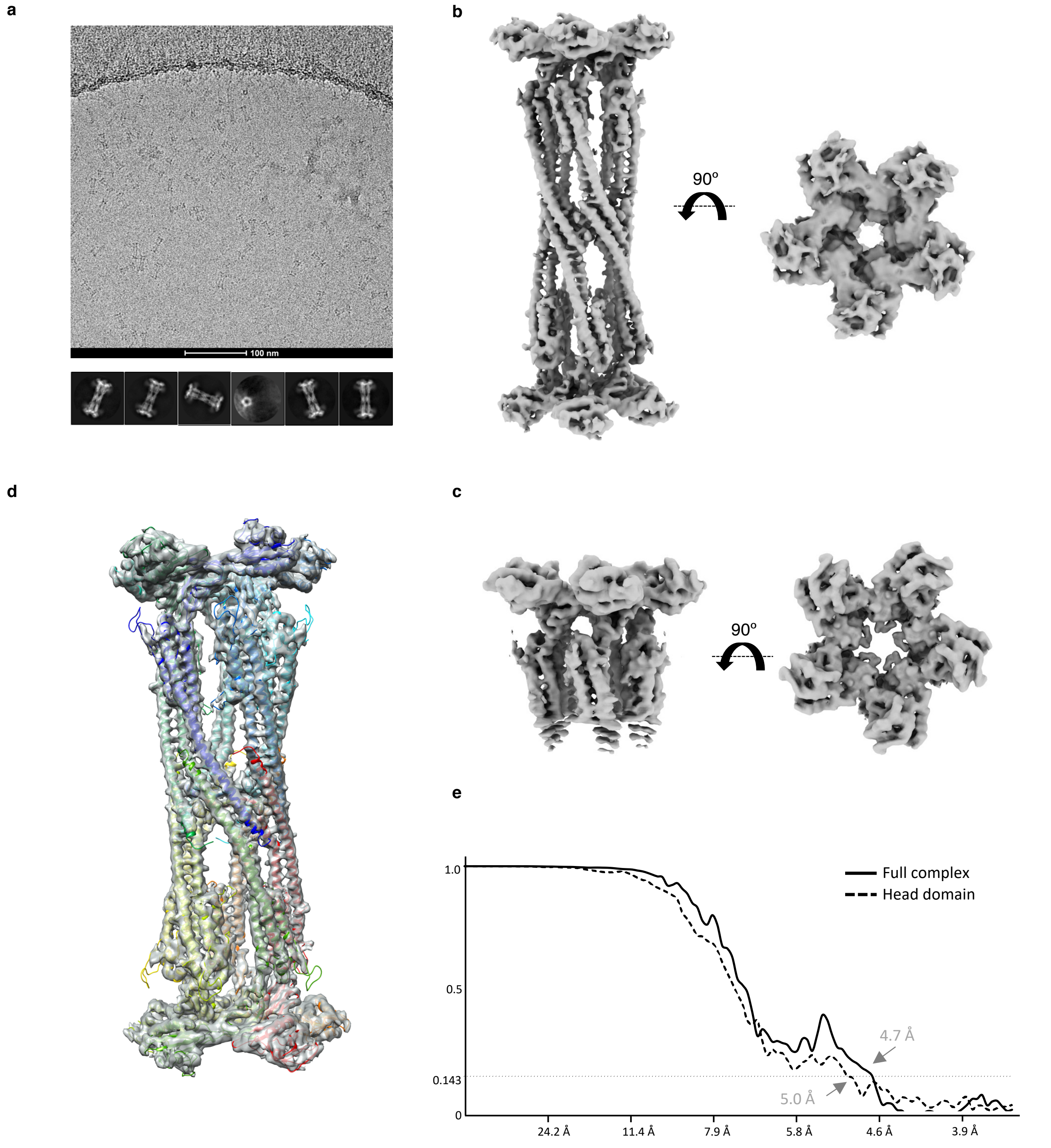

Supplementary Figure 2: Cryo-EM data for FliD$_{cj}$ flagellum capping protein. (a) Cryo-electron micrograph of the FliD$_{cj}$ complex. Large particles (~ 30 nm x 5 nm) are visible. Below are 2D classes generated from ~56000 particles. **(b)** Cryo-EM map of the full complex. Side view (left) and top view (right). **(c)** Cryo-EM map of the complex obtained from a masked refinement of (b) allowing for better resolution in the head domains. **(d)** Model of FliD$_{cj}$ built into the density map in (b). **(e)** FSC plots for the maps in (b) and (c) showing the resolution to be 4.7 Å and 5.0 Å respectively.).

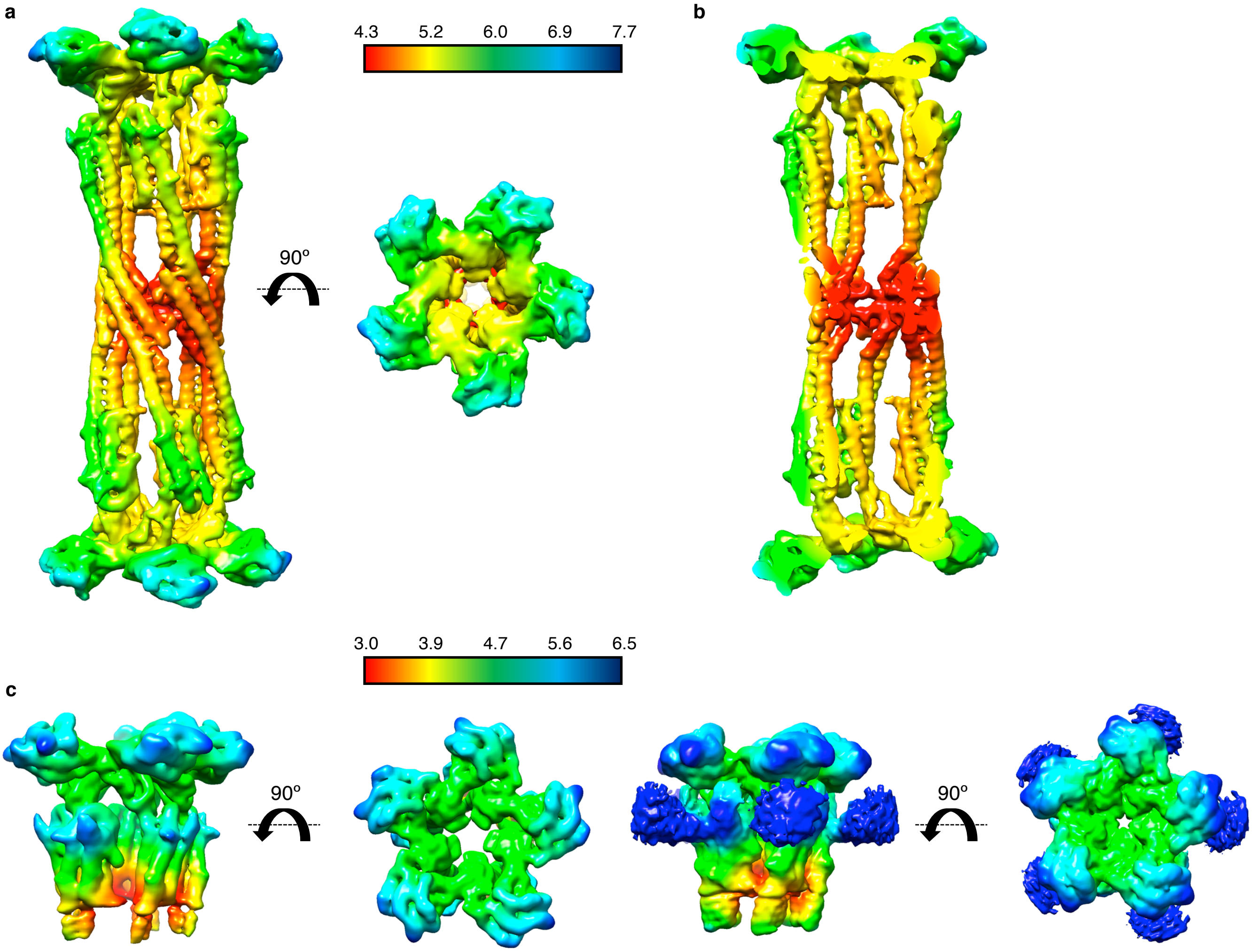

Supplementary Figure 3: Local resolution maps for FliD$_{cj}$. (a) Local resolution map for Decamer FliD$_{cj}$ (Supplementary Figure 2b) showing the uneven resolution distribution. Side view (left) and top view (right). The colour to resolution scale is shown above. **(b)** Cross section view of map in (a) with the colour to resolution scale as shown in (a). **(c)** Local resolution map for pentamer head domain FliD$_{cj}$ (Supplementary Figure 2c) showing the uneven resolution distribution. Side view (left) and top view (right). To the right is the local resolution map of the low-contour rendering of the head domain-focused cryo-EM map (Figure Supplementary Figure 2c) with the D4 domain at a low resolution (6.5 Å). Side view (left) and top view (right). The colour to resolution scale is shown above.

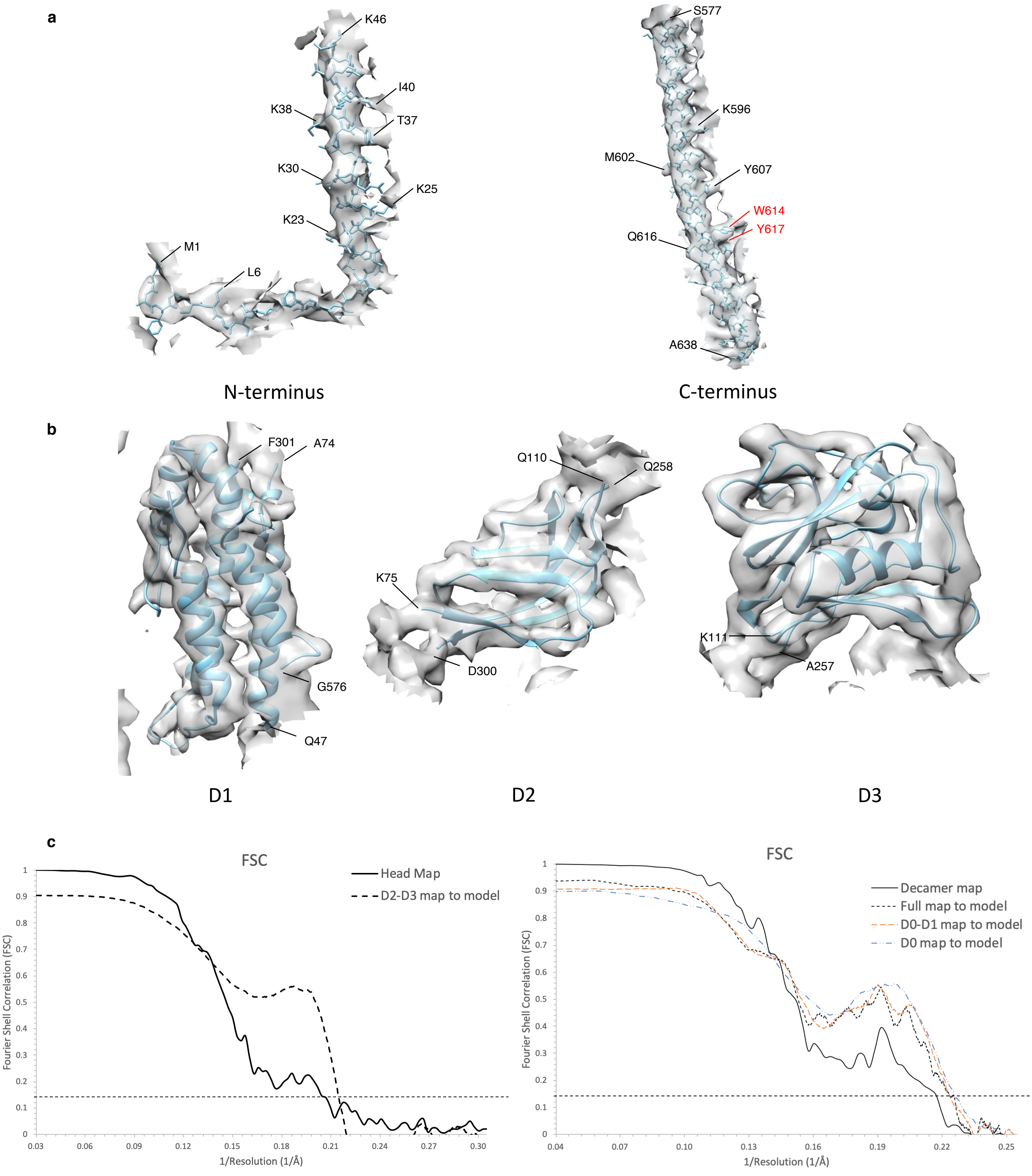

Supplementary Figure 4: Fit of the various regions of FliD$_{cj}$ into the cryo-EM map. (a) The N- and C- termini were manually built into the density in Coot by inserting a helix into the density and mutating the residues to the ones in the sequence. The helical register was determined by fitting large residues to their corresponding densities and examples of such are shown with black labels. The red labels correspond to co-varying residues shown in Supplementary Table 3. **(b)** D1, D2 and D3 domains were modelled in PHYRE2 (see Materials and Methods) into the density of map in figure Supplementary Figure 2b (for D1) and Supplementary Figure 2c (for D2 and D3). The start and end residues for each domain as well as their locations are labelled. **(c)** Map to model FSC maps for different domains of FliD$_{cj}$. On the left graph the resolution of the map of the head domain in Supplementary Figure 2c (5.0 Å) is compared to the modelled density of D2-D3 domains at the 0.143 FSC cut off point (4.8 Å). On the right graph the resolution of the map of the full decamer in Supplementary Figure 2b (4.7 Å) is compared to the full model (4.6 Å), D0-D1 leg domains (4.6 Å) and D0 terminal regions (4.5 Å).

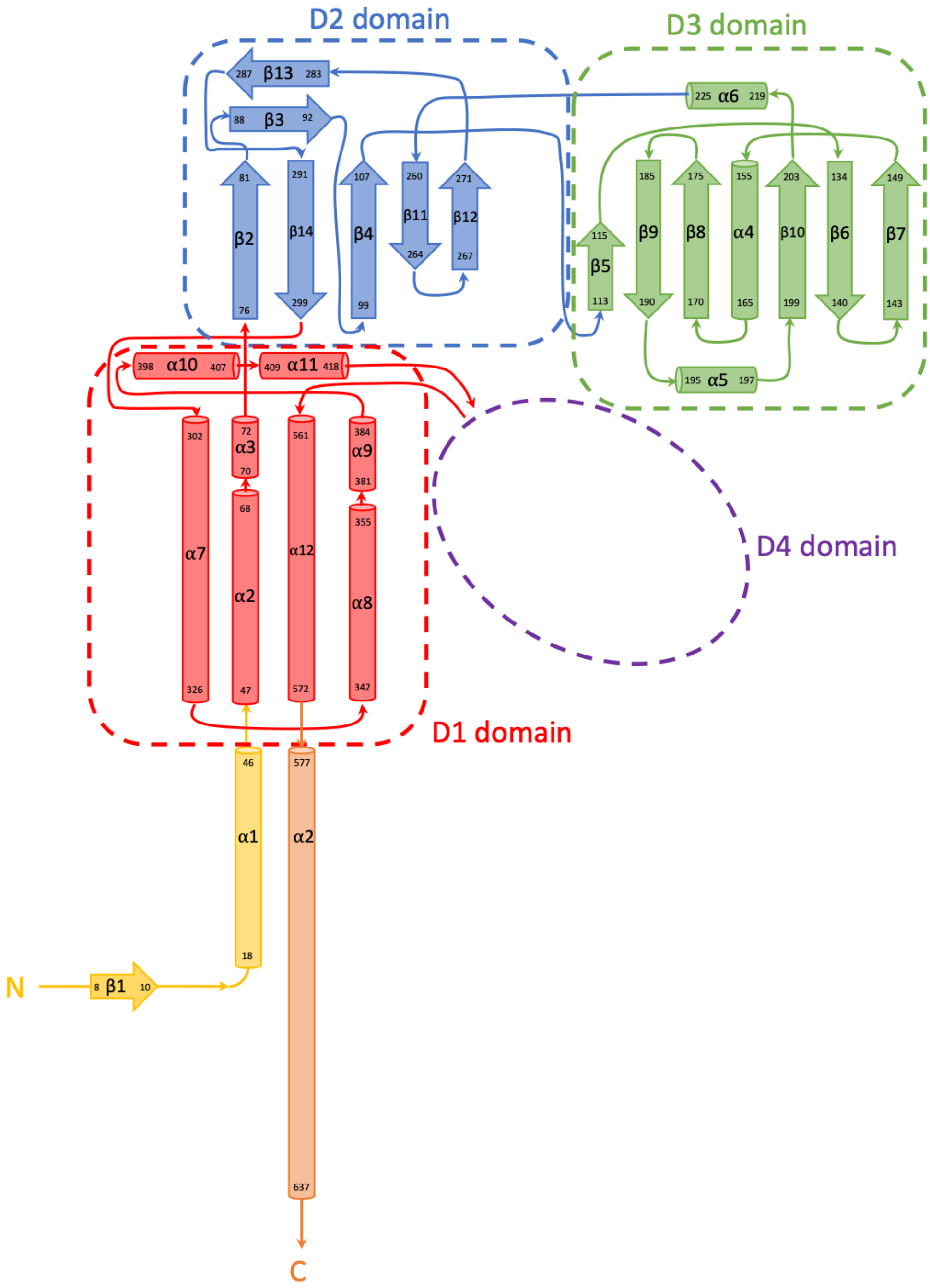

Supplementary Figure 5: Topology map of FliD$_{cj}$. Map was drawn from the structure built in Figure 1 (b).

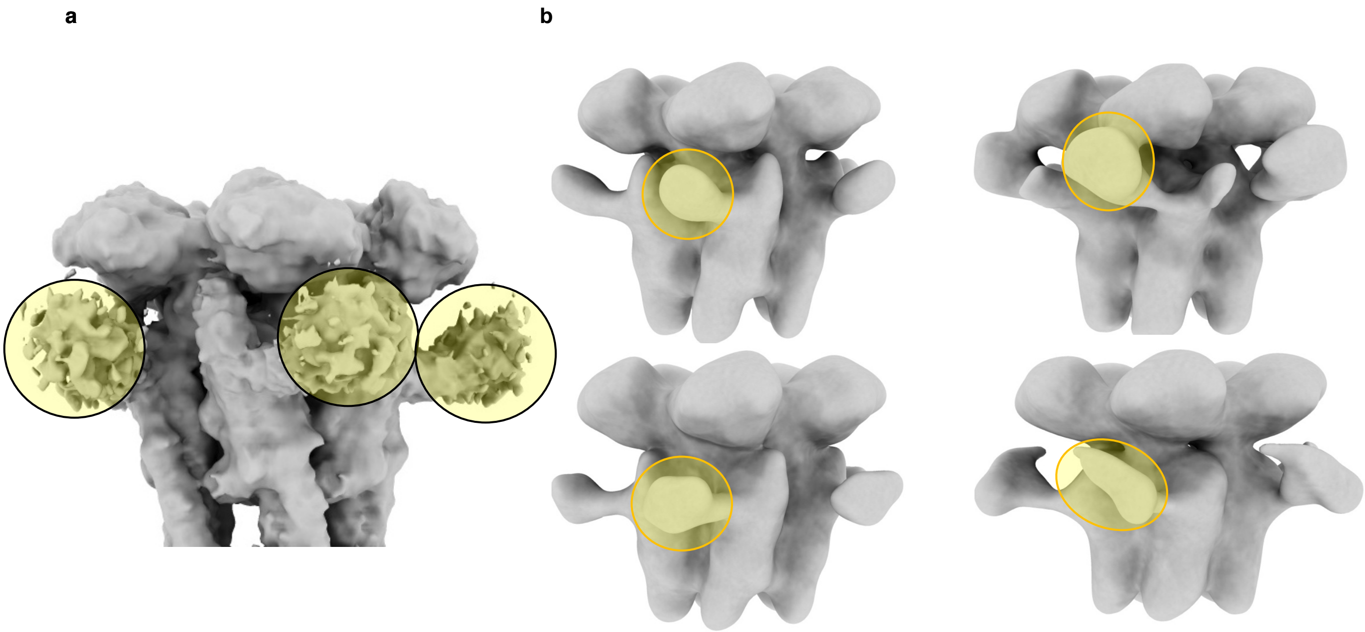

Supplementary Figure 6: FliD$_{cj}$ specific D4 domain. (a) Low-contour rendering of the head domain-focused cryo-EM map (Supplementary Figure 2c). Density attributed to the D4 domain is indicated with yellow circles. **(b)** Further 3D classification revealed distinct conformations of the D4 domain but remained at low resolution because of the low number of particles in each class.

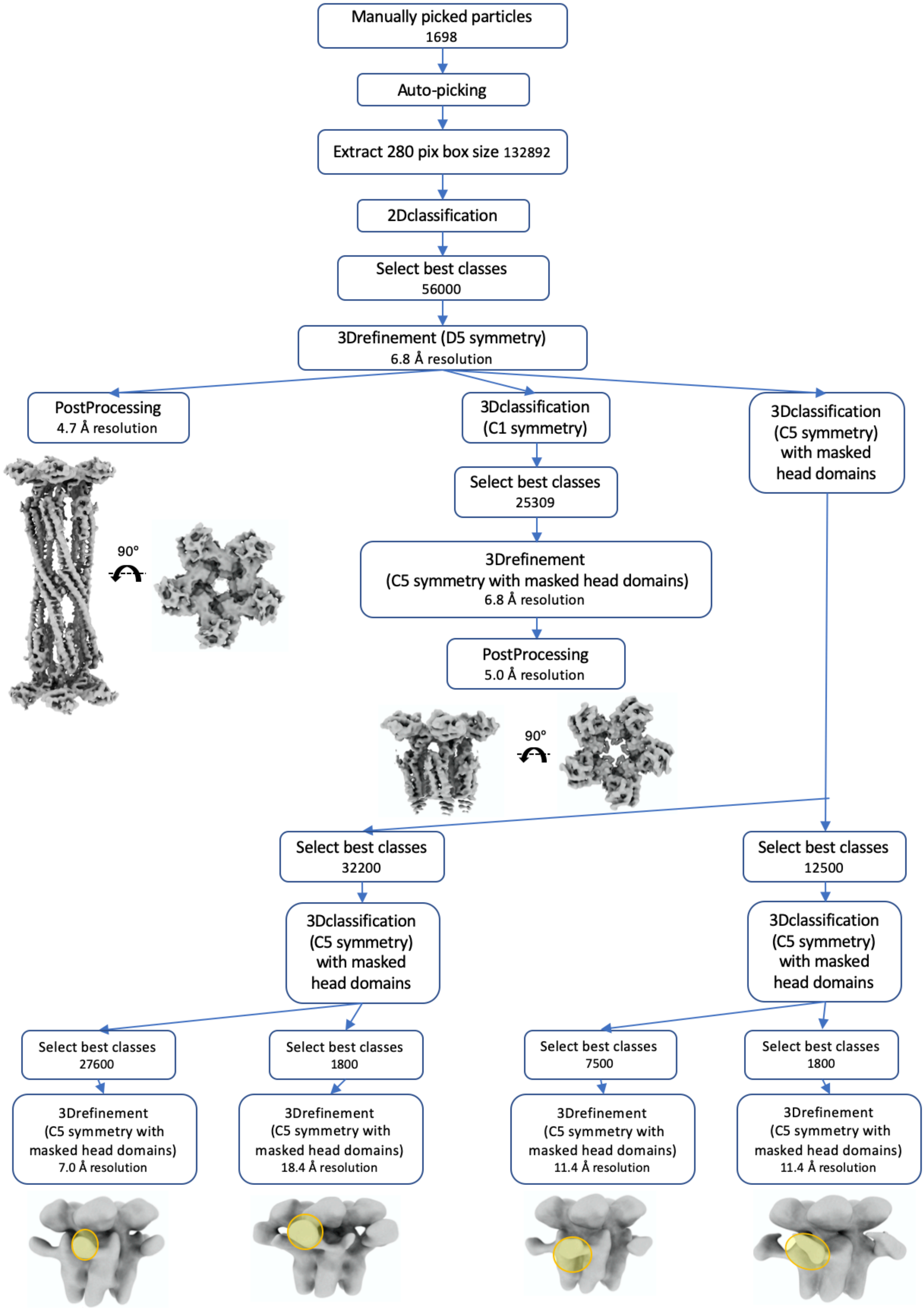

Supplementary Figure 7: Workflow of FliD$_{cj}$ processing in Relion. Maps presented in this figure correspond to Supplementary Figures 1b,c and Supplementary Figure 6c.

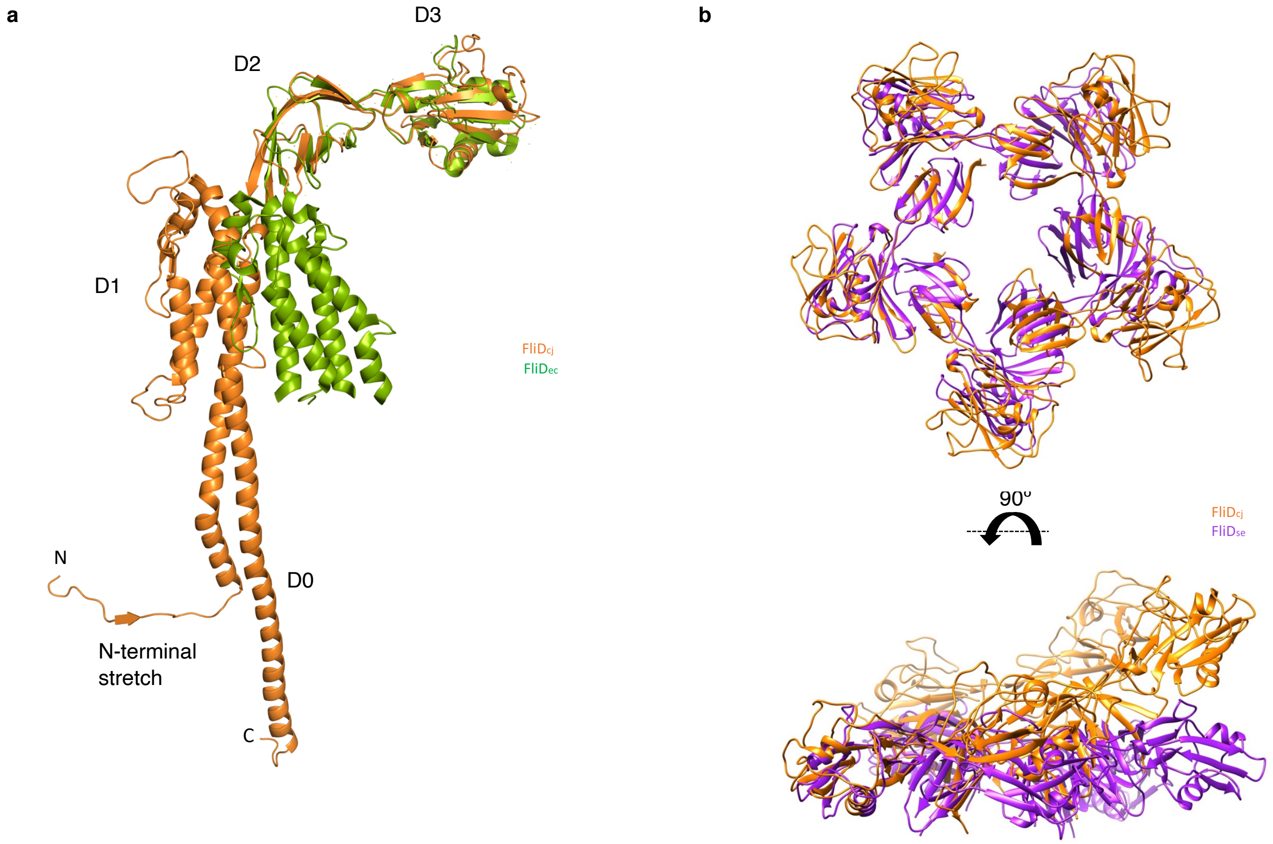

Supplementary Figure 8 : Comparison of the FliD structures across bacterial species. (a) Overlay of the FliD structures from *C. jejuni* ($FliD_{cj}$, this study, orange) and *E. coli* ($FliD_{ec}$, 5H5V, green). **(b)** Alignments of X-ray crystallography derived oligomeric structure of the D2-D3 domains from $FliD_{se}$ to that of the $FliD_{cj}$ pentamer. It is aligned using Chimera MatchMaker and $FliD_{cj}$ was aligned to Chain A of $FliD_{se}$. The angle at which the D2 and D3 domains interact are different with the $FliD_{se}$ being more planar than $FliD_{cj}$.

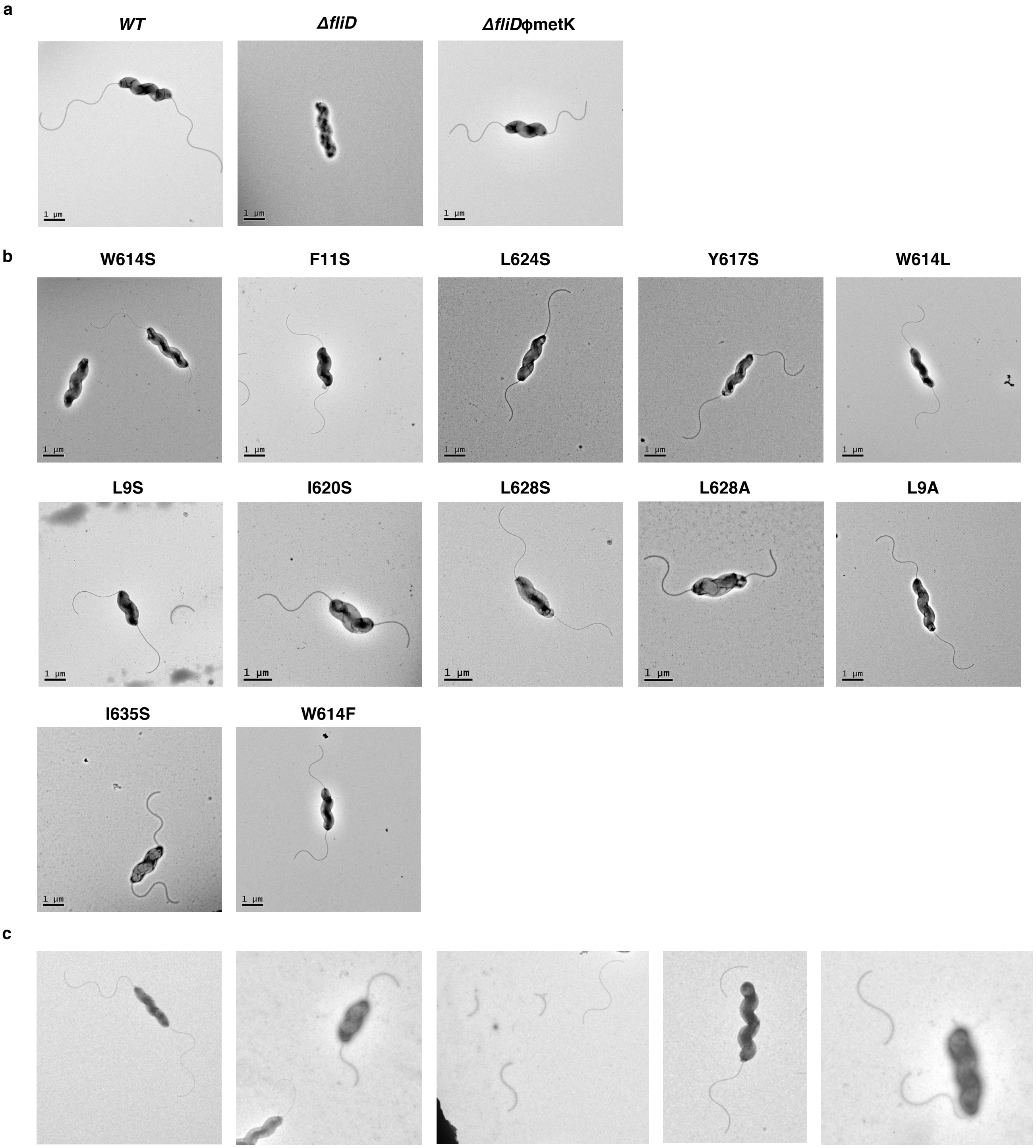

Supplementary Figure 9: Point mutant motility and flagella attachment. (a) Negative stain micrographs of cultured *C. jejuni* bacteria from the wild type, *fliD* knockout and complement mutant strains. **(b)** Negative stain micrographs of *C. jejuni* cultures containing FliD point mutants as in in Figure 3. **(c)** Illustrations of brittle filaments observed in our samples. From left to right: fully attached flagella, short stubby flagella with less than 2 inflection points, cut off flagella and fragments, flagellum cut off at the base, snapped off flagellum not at the base but further along the filament.

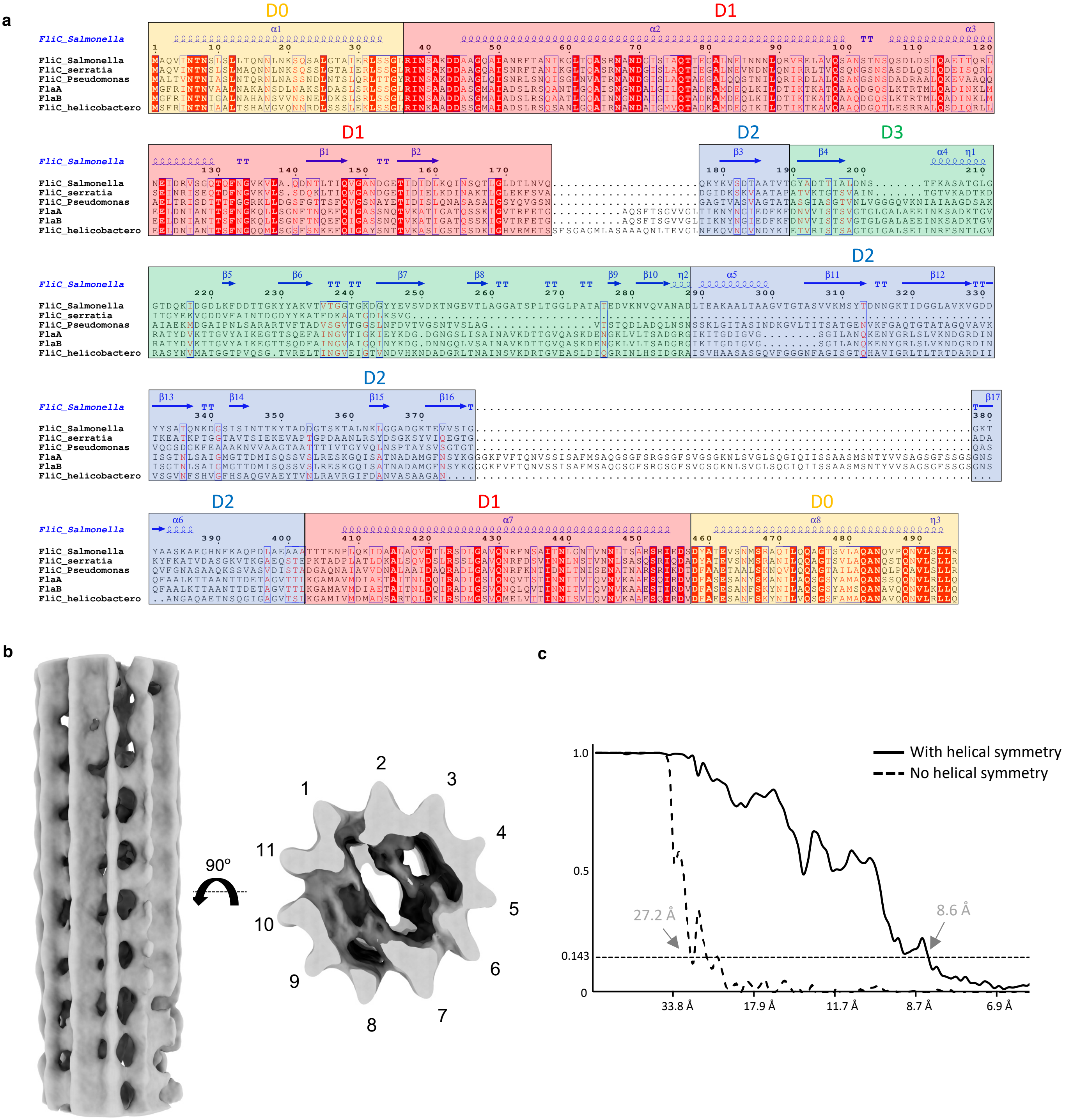

Supplementary Figure 10: Cryo-EM data for native *C.jejuni* flagella. (a) Sequence alignment for FliC from different species. The domains are colour coded as follows: *Yellow*: D0 terminal domains. *Red:* D1 domain. *Blue:* D2 domain. *Green:* D3 domain. **(b)** Asymmetric reconstruction of the *C.jejuni* native flagellum filament, with 11 protofilaments visible in the top view. Top view (right) and side view (left). **(c)** FSC curve for the 3D reconstruction of the *C. jejuni* flagellum filament, with (full line) and without (dotted line) helical symmetry.

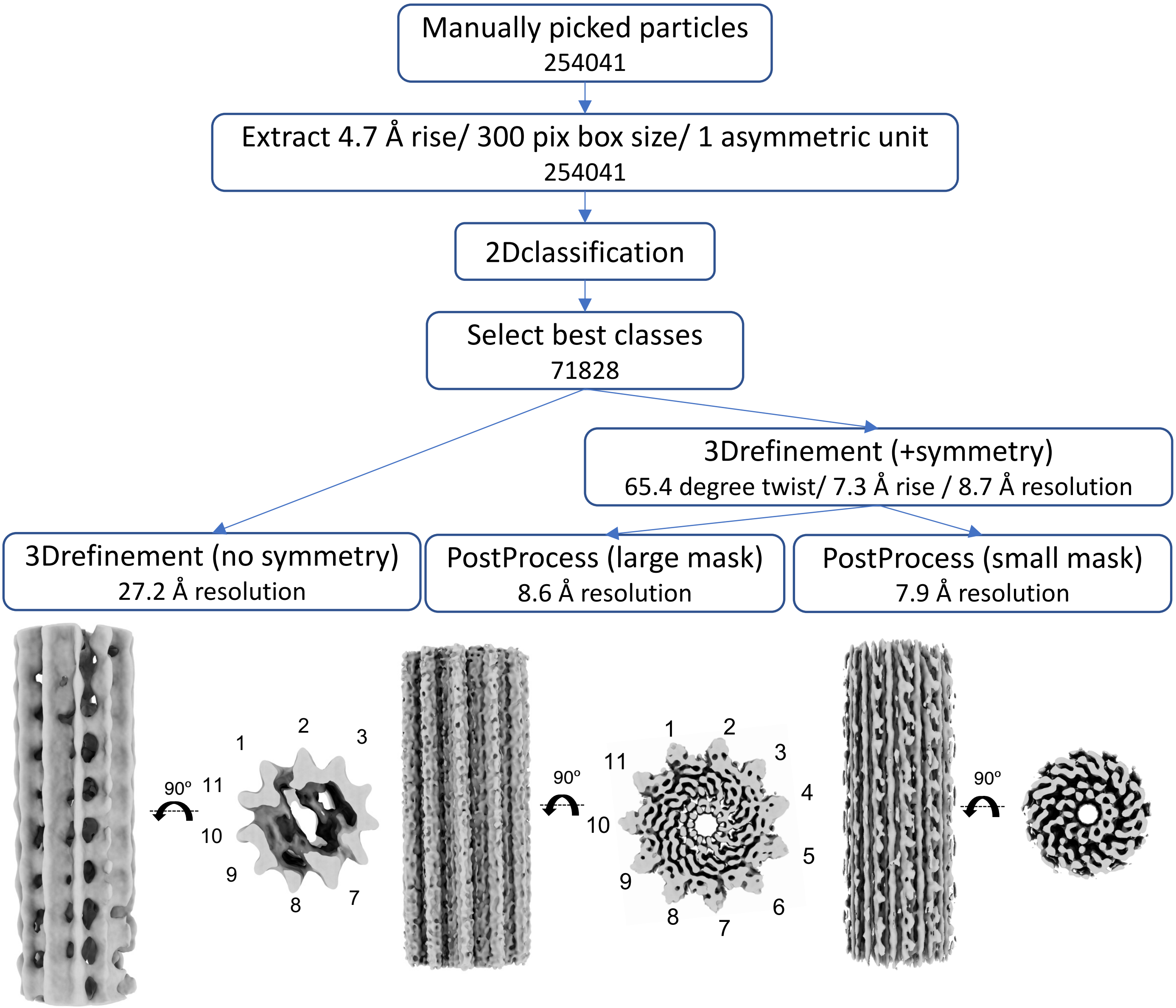

Supplementary Figure 11: Workflow of Flagellar filament processing in Relion. Maps presented in this figure correspond to Figure 4b and Supplementary Figure 10b.

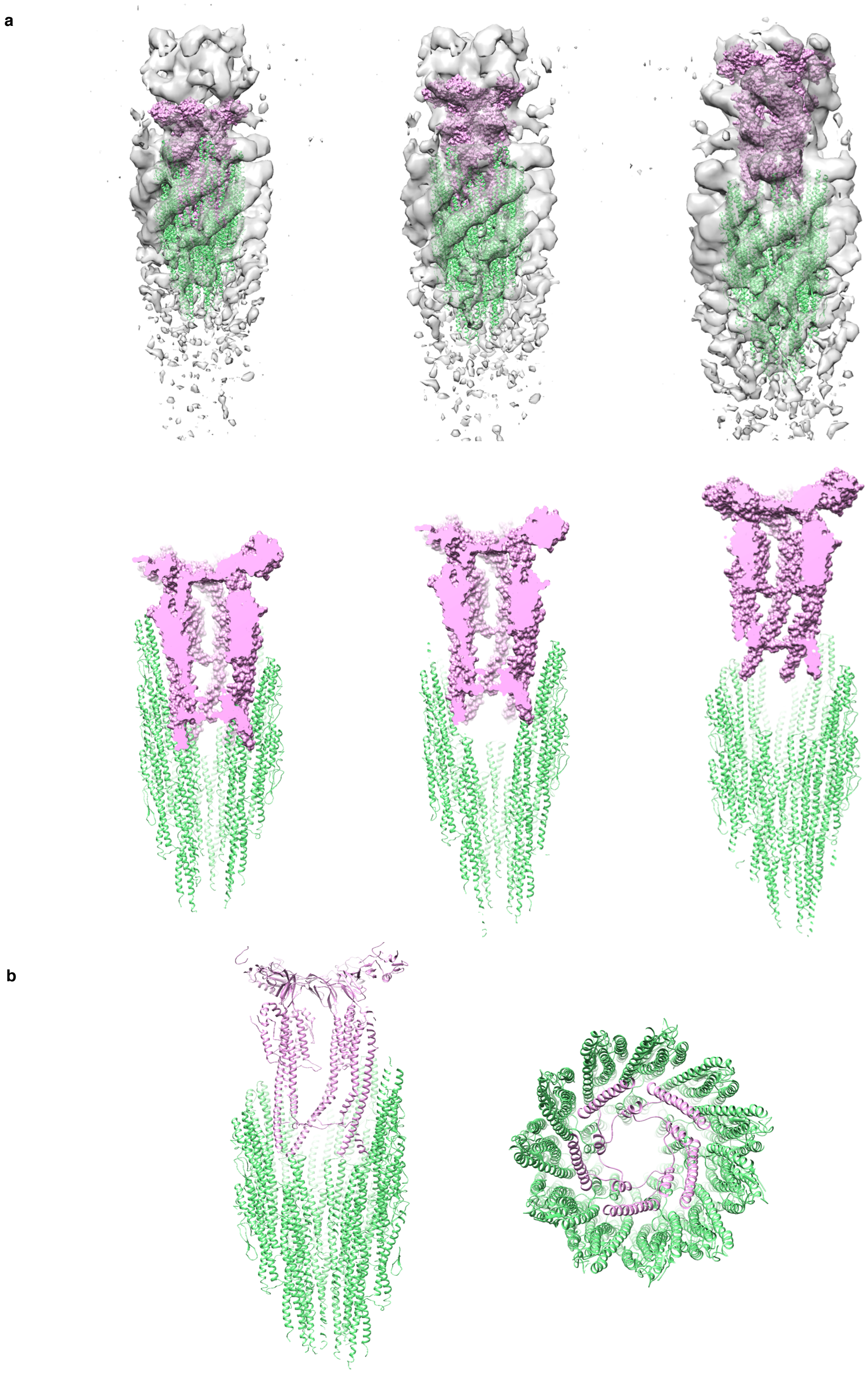

Supplementary Figure 12: Generation of FliD-filament complex model. (a) The 3 different fits of $FliD_{cj}$ and the filament to the tomography map of *B.burgdorferi* [1]. Under each fit there is a cross section of the models illustrating the overlap of each fit. **(b)** The middle fit model in (a) which was used as a basis for the proposed mechanism in Figure 5.

Name	Sequence 5' – 3'
fliDmutantF1	GAGCTCGGTACCCGGGGATCCTCTAGAGTCgtcgatataagcttttaactagc
fliDmutantR1	AAGCTGTCAAACATGAGAACCAAGGAGAATgtaatttagttttgatttctgttaa
fliDmutantF2	GAATTGTTTTAGTACCTAGCCAAGGTGTGCatacctctaaagactcaactcag
fliDmutantR2	AGAATACTCAAGCTTGCATGCCTGCAGGTCactgtttcattgttatgcac
KanF	ATTCTCCTTGGTTCTCATGTTTGACAGCTTAT
KanR	GCACACCTTGGCTAGGTACTAAAACAATTCAT
fliDcompF	AATATTCGTCTCACATGgcatttggtagtctatctagttta
fliDcompR	AATATTCGTCTCACATGgcttgattttgagaataagc

Supplementary Table 1: Primers for construction of *fliD* deletion mutant and complemented strains. The uppercase sequences of the *fliD* mutant primers are the adaptor regions used in the Gibson assembly cloning, while the lowercase sequences are the regions annealing to a region upstream of fliD (F1) and just inside the *fliD* coding region (R1) or at the end of the *fliD* coding region (F2) and downstream of *fliD* (R2). The KanF and KanR primers are adaptors that also amplify the kan gene from pJMK30

Name	Sequence 5' – 3'
F3L_F	TCATGGCATTaGGTAGTCTATC
F3S_F	TCATGGCATcTGGTAGTCTATC
F3_R	AAAAGTCCTTTCATTTAAAATGAAC
L9A_F	TCTATCTAGTgcAGGATTTGGTTC
L9S_F	TCTATCTAGTTcAGGATTTGGTTC
L9_R	CTACCAAATGCCATGAAAAAG
F11L_F	GTTTAGGATTaGGTTCTGGGG
F11S_F	GTTTAGGATcTGGTTCTGGGG
F11_R	TAGATAGACTACCAAATGC
Y315L_F	GGTGGATGCTctTAATGATTTAGTAAC
Y315S_F	GGTGGATGCTTcTAATGATTTAGTAAC
Y315_R	AAATCTTGCATGGCTTTTG
N316S_F	GATGCTTATAgTGATTTAGTAACCAATC
N316L_F	GATGCTTATctTGATTTAGTAACCAATC
N316_R	CACCAAATCTTGCATGGC
L318A_F	TTATAATGATgcAGTAACCAATCTTAATGC
L318S_F	TTATAATGATTcAGTAACCAATCTTAATGC
L318_R	GCATCCACCAAATCTTGC
L338A_F	AAAAGGAACTgcACAAGGCATC
L338S_F	AAAAGGAACTTcACAAGGCATC
L338_R	GTTCCAGTTTCACTATTATAG
D397N_F	TTTGAGTTTTaATTCTTCTAAATTTGAAC
D397L_F	TTTGAGTTTTctTTCTTCTAAATTTGAAC
D397_R	GTGCCTGCATCATTTAAAC
K400S_F	GATTCTTCTAgtTTTGAACAAAAAGTTAAAGAAGATC
K400L_F	GATTCTTCTttATTTGAACAAAAAGTTAAAGAAGATC
K400_R	AAAACTCAAAGTGCCTGC
L592A_F	TATTAAATCAgcAAATACCTCTAAA
L592_R	TCATTTGTCAAACTCTCATC
M602L_F	AACTCAGGCTcTGATTGATACAAG
M602_R	GAGTCTTTAGAGGTATTTAATG
W614L_F	GCGAATCAATtGTTGCAATATG
W614S_F	GCGAATCAATcGTTGCAATATG
W614F_F	GCGAATCAATtcTTGCAATATG
W614_R	CATTGTATCATATCTTGTATCAATC
Y617L_F	GGTTGCAATtaGAGAGTATTTTAAATAAAC
Y617S_F	GGTTGCAATcTGAGAGTATTTTAAATAAAC
Y617_R	ATTGATTCGCCATTGTATC
I620A_F	ATATGAGAGTgcTTTAAATAAACTCAATCAACAGC
I620S_F	ATATGAGAGTtcTTTAAATAAACTCAATCAACAGC
I620_R	TGCAACCATTGATTCGCC
L624A_F	TTTAAATAAAgcCAATCAACAGCTAAATACTGTAAC
L624S_F	TTTAAATAAAtcCAATCAACAGCTAAATACTGTAAC
L624_R	ATACTCTCATATTGCAACC
L628A_F	CAATCAACAGgcAAATACTGTAACTAATATG
L628S_F	CAATCAACAGtcAAATACTGTAACTAATATG
L628_R	AGTTTATTTAAAATACTCTCATATTG
I635A_F	AACTAATATGgcTAATGCGGCAAACAATTC
I635S_F	AACTAATATGtcTAATGCGGCAAACAATTC
I635_R	ACAGTATTTAGCTGTTGATTG

Supplementary Table 2: Primers for construction of *fliD* point mutants in *C.jejuni*. The forward primers (F) contain the mutated base(s) shown in lower case.

$FliD_{cj}$	$FlaA_{cj}$	Probability
Val631	Ala14	0.610818
Val631	Ser18	0.537091
Asn629	Ser18	0.484225
Ile635	Ala11	0.459525
Leu621	Leu25	0.439240
Ile635	Ala14	0.425490
Met634	Ala14	0.413679
Ala638	Phe3	0.400955
Leu621	Leu29	0.387116
Tyr617	Leu32	0.377176
Asn629	Ala22	0.375285
Gly340	Val9	0.371620
Ile635	Lys15	0.368281
Leu624	Asn21	0.352808
Trp614	Leu32	0.349173
Leu621	Leu25	0.348483
Leu24	Ile471	0.335947
Val631	Lys15	0.322094
Ser417	Gln487	0.321698
Asn643	Met1	0.321136
Asn629	Asn21	0.317865
Glu28	Ala45	0.317778
Asp390	Lys87	0.314706

Supplementary Table 3: Co-evolution analysis between aligned FliD and FlaA (flagellin) sequences across bacterial species performed using RaptorX ComplexContact server.

Protein	Codon optimized DNA sequence
FliD$_{cj}$	GCCTTCGGTAGCCTGAGCAGCCTGGGCTTCGGTAGCGGCGTTCTGACCCAGGATACCATCGATAAACTGAAAGAAG<br>CGGAACAGAAAGCTCGCATCGACCCGTATACCAAGAAAATCGAAGAAAACACCACCAAACAGAAAGATCTGACCGA<br>AATTAAAACCAAACTGCTGTCTTTCCAGACCGCGGTTTCCTCCCTGGCGGACGCGACCGTGTTCGCGAAACGTAAA<br>GTGGTTGGCAGCATCAGCGATAACCCGCCGGCAAGCCTGACCGTTAACTCTGGTGTGGCGCTGCAGTCCATGAACA<br>TTAACGTGACCCAGCTGGCTCAGAAAGATGTGTATCAGTCCAAAGGTCTGGCGAACGACAGCGGTTTTGTTAACGCT<br>AACCTGACCGGCACCACCGATCTGACCTTCTTCTCTAACGGCAAAGAATACACCGTGACCGTGGACAAAAACACCAC<br>CTACCGTGATCTGGCGGATAAAATCAACGAAGCTAGCGGCGGTGAAATCGTTGCGAAAATCGTAAACACGGGCGAA<br>AAAGGCACCCCGTACCGTCTGACCCTGACCTCCAAAGAAACCGGCGAAGATAGCGCGATCTCCTTCTACGCGGGTA<br>AAAAAGACGCGCAGGGTCAGTACCAGAGCGACCCGGAGGCGGAAAACATCTTCTCCAACCTGGGCTGGGAACTGG<br>ACAAAACCACCCAGACCATCGACCCGGCAAAAGATAAGAAAGGCTACGGCATTAAAGATGCGTCTCTGCACATCCA<br>GACCGCGCAGAACGCGGAATTTACCCTGGACGGTATCAAAATGTTCCGTTCCTCCAACACCGTGACCGACCTGGGT<br>GTTGGTATGACCCTGACCCTGAACAAAACCGGCGAAATCAACTTCGATGTTCAGCAGGACTTCGAAGGTGTTACCAA<br>AGCCATGCAGGACCTGGTAGATGCTTATAACGATCTGGTTACCAACCTGAACGCGGCGACCGATTACAACAGCGAA<br>ACCGGCACCAAAGGCACCCTGCAGGGCATTTCTGAAGTTAACAGCATCCGTAGCTCTATTCTGGCGGATTTGTTCGA<br>CTCTCAGGTGGTTGACGGTACCACCGAAGATGCTAACGGTAACAAAGTTAACACCAAAGTTATGCTGTCCATGCAGG<br>ACTTCGGCTTATCTCTGAACGATGCGGGCACCCTGAGCTTCGACTCTTCCAAATTCGAACAGAAAGTTAAAGAAGAT<br>CCGGATTCTACCGAATCCTTCTTTTCTAACATCACTAAATACGAAGATATTAACCACACCGGTGAAGTGATCAAACAG<br>GGCAGCCTGAACCAGTACCTGGATAGCAGCGGTACCGGCAACAAAGGTCTGGATTTCAAACCAGGCGACTTCACCA<br>TCGTTTTCAACAACCAGACCTATGACCTGTCCAAAAATAGCGACGGCACCAACTTCAAGCTGACCGGTAAAACCGAA<br>GAAGAATTGCTGCAAAACCTGGCAAACCACATCAACTCAAAAGGTATCGAAGGTCTGAAAGTTAAAGTGGAATCGTA<br>CGATCAGAACGGCGTGAAAGGTTTCAAGCTGAACTTCTCTGGTGATGGTAGCTCTGATTTCTCTATTAAAGGCAACG<br>CGACCATCTTGCAGGAACTGGGCCTGTCTGATGTTAATATCACCTCCAAACCGATCGAAGGCAAAGGCATCTTCTCC<br>AAACTGAAAGCTACCCTGCAGGAAATGACCGGTAAAGACGGTAGCATCACCAAATATGATGAAAGCCTGACCAATGA<br>CATTAAGTCACTGAACACTAGTAAAGATAGCACGCAGGCAATGATTGACACCCGCTATGATACCATGGCTAACCAAT<br>GGCTGCAGTACGAATCGATCCTGAATAAACTGAACCAGCAGCTGAATACCGTGACTAACATGATTAACGCAGCTAAT<br>AATAGTAATAACTAATAA
FliD$_{pa}$	ATGGCGGGTATCTCTATCGGCGTTGGTTCTACCGACTACACCGACCTGGTTAACAAAATGGTTAACCTGGAAGGTGC<br>GGCTAAAACCAACCAGCTGGCTACCCTGGAAAAAACCACTACCACCCGTCTGACCGCTCTGGGTCAGTTCAAATCT<br>GCGATTTCTGCTTTCCAGACCGCTCTGACCGCGCTGAACTCTAACGCGGTGTTCATGGCTCGTACCGCTAAATCTTC<br>CAACGAAGATATCCTGAAAGCGTCTGCTACCCAGTCTGCAGTTGCTGGTACTTACCAGATCCAGGTTAACTCTCTGG<br>CGACCTCTTCTAAAATCGCGCTGCAGGCGATCGCTGATCCGGCGAACGCTAAATTCAACTCCGGTACTCTGAACATC<br>TCCGTTGGCGACACCAAACTGCCGGCGATTACTGTTGATTCTTCTAACAACACTCTGGCGGGTATGCGTGATGCAAT<br>CAACCAGGCGGGTAAAGAAGCTGGCGTTTCTGCTACCATCATCACTGACAACTCCGGCTCTCGTCTGGTTCTGTCTT<br>CTACCAAAACTGGTGATGGTAAAGACATTAAAGTTGAAGTCTCTGATGACGGTTCTGGTGGTAACACCTCTCTGTCC<br>CAGCTGGCTTTCGACCCGGCAACCGCGCCGAAACTGTCCGATGGTGCAGCTGCAGGCTATGTTACTAAAGCTGCTA<br>ACGGTGAAATCACTGTTGATGGTCTGAAACGTTCTATCGCTTCCAACAGCGTATCTGACGTTATTGACGGTGTTTCCT<br>TCGATGTGAAAGCAGTTACCGAAGCAGGTAAACCAATCACTCTGACCGTTTCTCGTGACGACGCAGGTGTTAAAGAT<br>AACGTTAAAAAATTCGTTGAAGCTTATAACACCCTGACCAAATTCATCAACGAACAGACTGTAGTTACTAAAGTTGGT<br>GAAGATAAAAACCCGGTTACTGGTGCACTGCTGGGTGACGCATCTGTGCGTGCTCTGGTTAACACCATGCGTAGCG<br>AACTGATCGCGTCTAACGAAAACGGTTCTGTTCGTAACCTGGCGGCTCTGGGTATCACTACCACTAAAGATGGTACC<br>CTGGAAATCGATGAGAAAAAACTGGACAAAGCTATTTCTGCTGATTTCGAAGGTGTTGCTTCCTACTTCACCGGTGA<br>CACCGGTCTGGCGAAACGCCTGGGTGACAAAATGAAACCGTACACCGACGCTCAGGGCATTCTGGATCAGCGTACC<br>ACCACTCTGCAGAAAACCCTGTCTAACGTTGATACCCAGAAAGCGGACCTGGCTAAACGTCTGGCGGCACTGCAGG<br>AAAAACTGACCACCCAGTTCAACCTGCTGTCTGCGATGCAGGACGAAATGACCAAACGCCAGAAATCTATCACCGAT<br>AACCTGGCGTCTCTGCCGTACGGCTCTGGTAAGAAAACCTAA
FliD$_{sm}$	ATGGCGACCATCTCTTCTCTGGGCCTGGGTTCTGGCCTGGACCTGAACGGTCTGCTGGATAAACTGACCAAAGCTG<br>AACAGCAGCGTCTGACCCCGTACACCACTAAACAGTCTTCTTATAACGCTCAGCTGACCGGTTACGGTACCCTGAAA<br>GGTGCGCTGGAAAAATTCGACAACCTGTCTAAAGAAATGGCAAAAGAAGATTTCTTCAAAGCTACTACCGCTACCGA<br>ACACGACGCGTTCAAAATCACCACCAACGCAAAAGCTGTTCCGGGTAACTACGTTGTGGAAGTTAAAAACCTGGCAC<br>AGGCTCAGACCCTGACCACCCAGGCGAAAGTTAGCGACCAGGGTGCTAAACTGGGTGCGGAAGGCGTGACCGATC<br>GTTCTCTGACCATCACCGCTGGTAACCCGCCGAAAGAAACCAAAATCCCGCTGTCTGACGATCAGACCAGCCTGGT<br>TGAACTGCGTGATGCAATTAACGGTGCGAAAGCTGGCGTTACCGCGTCTATTATGCGTGTGGGTGATAACGACTAC<br>CAGCTGGCGGTTTCTTCTTCTACCACCGGTGAAAACAACAAAATTAGCCTGCAGGTTGATAACGATGATAAACTGGG<br>TGACATCCTGAACTACAACGCGACTCGTGGTACCTCCACTGCTATGAAACAGACTGTTGCTCCGCAGGACGCGGAA<br>CTGATGGTTAACGGCACCGCGATCAAACGTTCTACCAACTCTATCTCTGATGCGCTGCAGGGTGTTACTATCGATCT<br>GAAAACCAAAACCAAAACCGATGAACCGCAGCACCTGGTGATTTCCACCAACACCGCGGGTACTACCGACAAAATC<br>AAAGAATGGGTTGACTCTTACAACTCTCTGCTGGACACCTTCAACGCACTGTCTAAATTCACCCCGGTTAAAACCGG<br>TGAAGCGCCGAACCCGTCTAACGGTCCGCTGCTGGGTGACAACACCCTGCGTGGCGTTCAGTCTTCCATCAAATCT<br>GCACTGTCTGCTGCGCAGGACAACCCGGAACTGAAAGGTCTGGGCAACCTGGGTATCTCTACCAACACTAAAACCG<br>GCAAACTGGAAATCGATTCTGCTAAACTGAAAAAAGCTATGGACGAAAAACCGGACCAGGTTTCTAACTTCTTCGTT<br>GGTAACGGTAAAGACACCGGTATGGCGACCGAAATCCACAACGAAATCCAGTCCTACATCAAAAGCGGCGGTATCA<br>TCGAAAACTCTACTAAATCTATCAACACCAACCTGGACCGCCTGAACTCTCAGATCACTACCGTTACTGCGTCTATCC<br>AGAACACCATCGACCGTTACAAACAGCAGTTCGTTCAGCTGGATACCATGATGTCTAAAATGAACGGTACCTCTAAC<br>TACCTGGCACAGCAGTTCAAATAA

Supplementary Table 4: Codon optimised DNA sequences of FliD$_{cj}$, FliD$_{pa}$ and FliD$_{sm}$ as provided by BioBasic.

Supplementary References:

1. Zhang, K. *et al.* Analysis of a flagellar filament cap mutant reveals that HtrA serine protease degrades unfolded flagellin protein in the periplasm of Borrelia burgdorferi. *Mol. Microbiol*. **111**, 1652–1670 (2019).
